# Pathophysiological changes that affect drug disposition in protein-energy malnourished children

**DOI:** 10.1186/1743-7075-6-50

**Published:** 2009-12-01

**Authors:** Kazeem A Oshikoya, Idowu O Senbanjo

**Affiliations:** 1 Pharmacology Department, Lagos State University College of Medicine, PMB 21266, Ikeja, Lagos, Nigeria; 2 Paediatrics Department, Lagos State University Teaching Hospital, Ikeja, Lagos, Nigeria; 3 Academic Division of Child Health, University of Nottingham, The Medical School, Royal Derby Children's Hospital, Uttoxeter Road, Derby DE22 3DT, UK

## Abstract

Protein-energy malnutrition (PEM) is a major public health problem affecting a high proportion of infants and older children world-wide and accounts for a high childhood morbidity and mortality in the developing countries. The epidemiology of PEM has been extensively studied globally and management guidelines formulated by the World Health Organization (WHO). A wide spectrum of infections such as measles, malaria, acute respiratory tract infection, intestinal parasitosis, tuberculosis and HIV/AIDS may complicate PEM with two or more infections co-existing. Thus, numerous drugs may be required to treat the patients. In-spite of abundant literature on the epidemiology and management of PEM, focus on metabolism and therapeutic drug monitoring is lacking. A sound knowledge of pathophysiology of PEM and pharmacology of the drugs frequently used for their treatment is required for safe and rational treatment. In this review, we discuss the pathophysiological changes in children with PEM that may affect the disposition of drugs frequently used for their treatment. This review has established abnormal disposition of drugs in children with PEM that may require dosage modification. However, the relevance of these abnormalities to the clinical management of PEM remains inconclusive. At present, there are no good indications for drug dosage modification in PEM; but for drug safety purposes, further studies are required to accurately determine dosages of drugs frequently used for children with PEM.

## Introduction

Protein-energy malnutrition (PEM) is generally a nutritional problem that results from varying proportions of protein and calorie deficiency in infants and young children of developing countries [1]. It is a global public health problem, affecting children from African, Asian, Latin American and Caribbean regions [2-4]. Approximately 70% of the world's malnourished children live in Asia [4]. PEM is directly or indirectly responsible for about half of the 10.8 million deaths per year in under five children in developing countries [5]. The major risk factors that can predispose a child to having PEM include poverty, lack of access to quality food, cultural and religious food customs, poor maternal education, inadequate breast feeding, and lack of quality healthcare [5-7]. In addition to macronutrient deficiency, there is clinical and/or subclinical deficiency of micronutrients [8].

The World Health Organization (WHO) has broadly classified PEM into underweight, wasting and stunting [9] based on the physical status and anthropometric measurements of a child in a given population. This method of classification is however more useful in epidemiological studies of PEM in a large population of children [3,10]. Other methods of PEM classification have been used in clinical studies [11]. Most of the previous studies that evaluated drug disposition in children with PEM have used the Gomez [12], Waterlow [13] and Wellcome Trust Party [14] classifications. However, the Wellcome Trust Party classification is widely used more frequently than others in clinical studies of PEM. Apart from using the anthropometric measurements; clinical symptoms and signs, and findings from laboratory investigations are additional criteria used in the classification PEM. Clinically, PEM is a disease spectrum that can present as underweight, marasmus, marasmic-kwashiorkor or kwashiorkor; the severe forms being marasmus, marasmic-kwashiorkor and kwashiorkor [8].

The pathophysiological changes in PEM are as a result of the imbalance between nutrient supplies and requirements. These changes account for the clinical signs and symptoms of PEM [15]. Protein deficiency and lack of immune mediators is responsible for immunologic deficiency in the humoral and cellular subsystem, which predisposes a child with PEM to infections [16]. Carbohydrate deficiency can lead to abnormal lipid metabolism which may manifest as marked reduction of adipose tissue in a child with marasmus [17]. Lack of a substrate (tyrosine amino acid) and coenzymes required for the synthesis of pigments in the hair and skin result in changes in the hair colour and hyperpigmentation of the skin in marsamic-kwashiorkor and kwashiorkor [18]. Presences of aflatoxins and protein deficiency have been implicated in the aetiology of oedema in kwashiorkor rather than due to severe anaemia that was postulated in the past [8,19].

PEM is usually associated with infections such as measles, acute respiratory tract infection, malaria, HIV/AIDS and tuberculosis [20]. The World Health Organization (WHO) has recommended empirical treatment of all children admitted for PEM with antibiotics because the signs and symptoms of infection may be masked [21]. However, other specific associated infections would require treatment with numerous drugs such as the more potent antibiotics, antimalarials, antiretroviral and anti-tuberculosis drugs. The disposition of these drugs has not been well studied in children with PEM.

Many of the physiological systems of children with PEM are deranged and either directly or indirectly influence the disposition of drugs frequently used for their treatment. This review was therefore aimed at discussing the various physiological derangements as they affect drug disposition in children with PEM.

### Body fluid distribution

The total body water (TBW) is increased in proportion to the degree of malnutrition [22]. Children with marasmus have the highest TBW but contrarily, there is a significant reduction in adipose mass as well as lean body mass in marasmus and marasmic-kwashiorkor which can alter the apparent volume of distribution of drugs [23]. The distribution into adipose tissue of lipid soluble drugs is known to be reduced in PEM [23]. A major clinical implication of such reduction is that the concentration of the lipid soluble drug would increase at the target tissues, thus prolonging their pharmacodynamic actions.

Body fluid compartments are altered by many factors such as nutritional status and disease [24]. The increased total body water is associated with a proportionate rise in extracellular fluid (ECF); this is seen particularly in children with oedema [25].

Infection is a major complication of PEM and may occur without the classical signs and symptoms. Consequently, the WHO has recommended that all children admitted with PEM should routinely receive parenteral antibiotics [21]. Tissue penetration of antibiotics is very important in the treatment of infections, since many infections are located in deep tissues, or more specifically in the extracellular tissue space [26]. Studies on antibiotics distribution into the body fluids have shown that serum levels do not necessarily reflect the concentration in deep tissues, which is frequently 50% less [27]. A direct relationship between an antibiotic dose (cefalotin) and the level achieved in the interstitial fluids has been established in a study [28]. The antibiotic concentrations in the serum and the interstitial fluids showed the same time-concentration response irrespective of the dose.

The volume of distribution is the most important parameter that determines the peak concentration of aminoglycosides and is closely related to the extracellular fluid volume because of the low level of protein binding and high solubility in water [29]. The volume of distribution of aminoglycosides is increased in oedematous states, burns (or extensive skin desquamation) and with capillary leak in severe infection. Children with severe PEM may have all the three complications: oedema, wide spread dermatosis and shock due to bacterial infection [30].

Oedema is a form of ECF seen in both kwashiorkor and marasmic-kwashiorkor. It has been shown that plasma protein concentration is often particularly low in children with oedema. The oedema may clear during nutritional rehabilitation without any change in serum albumin concentration [31].

The bioavalaibility of penicillin [32,33], tobramycin [25], gentamicin [34], streptomycin [35] and cefoxitin [36] after intramuscular (i.m) administration did not significantly differ in children with kwashiorkor when compared with normal and nutritionally rehabilitated children. Therefore i.m administration of antibiotics to children with oedematous PEM (marasmic-kwashiorkor or kwashiorkor) may be therapeutically acceptable. However, absolute gross oedema (greater than 30% of body weight) and shock may reduce bioavailability of i.m antibiotics in children with kwashiorkor [37].

### Plasma proteins

Following absorption and entry of drugs into the vascular system, drug molecules frequently bind to plasma proteins. In general, acidic drugs bind to albumin and basic drugs bind to α1-acid glycoprotein [38]. This is generally reversible and a state of equilibrium is maintained between bound and unbound drug. Drugs that are protein bound do not pass through cell membranes; only the unbound (free) fraction can distribute into the tissue compartment [39]. Once in the extravascular space, drug activity is variable. Drugs may be metabolised or bind to tissue receptors resulting in a pharmacologic, toxic, or neutral effect [40].

Hypoproteinaemia is a common feature of PEM [41,42]. Plasma albumin and fractions of the glycoproteins responsible for binding drugs are decreased [43-45]. As a result of this decreased protein binding, in theory, there may be a substantial increase in the plasma free-drug fractions of highly protein-bound drugs and children with PEM may experience variations in their response to drug treatment or be at risk of increased drug toxicity [46]. However, in clinical practice, the decreased plasma protein has not been reported to significantly increase the plasma free-drug fractions in children with PEM.

### Infection and immunity

Malaria, bronchopneumonia and measles are most frequently associated with malnutrition in African children. In addition, urinary and gastrointestinal tracts infections, as well as septicaemia may complicate PEM [41,47]. Many of these infections are due to Gram positive and Gram negative organisms and are usually implicated in patients with severe PEM [48-50].

A study has shown that confirmed bacteraemia occurred in 12% of severe PEM on admissions and complicated 26% of case fatalities with 52% of the deaths occurring within 48 hours of admission in-spite of treatment with antibiotics recommended by the WHO [30]. The in vitro antibiotic susceptibility testing indicated that 85% of the organisms causing the bacteraemia were susceptible to ampicillin and gentamicin recommended by the WHO [21]. Another study that examined susceptibility patterns among Gram-negative bacilli reported lower levels of isolates fully susceptible to gentamicin (76%); however, in children with PEM, gentamicin resistance was not found to be associated with mortality [51]. These deaths upon adequate treatment of the infection in PEM was attributed to additional risk factors that may be associated with in-hospital deaths, which may include alterations in the pharmacokinetics of the drugs [30]. The clinical efficacy and toxicity potentials of antibiotics are determined by their penetration to superficial and deep tissues; therefore, knowledge of pharmacodynamics and tissue distribution principles is essential for the rational management of antimicrobial agents in children with PEM [52].

### Changes in gastrointestinal system

Diarrhoea and vomiting are common problems of PEM. Therefore oral drugs may not be retained, and if it is, the transit time through the bowel may be decreased. PEM is associated with various degrees of intestinal malabsorption [53,54]. Previous work of the Institute of Nutrition of Central America and Panama (INCAP) has shown that, in general, the malabsorptive state disappears as the child recovers [55], but some of the morphological changes persist despite improved nutritional status [56]. However, the persistent morphological changes after nutritional rehabilitation are not associated with any degree of malabsorption. PEM is associated with villous atrophy of the jejunal mucosa [41,56,57] and this may impair drug absorption [12]. The oral absorption of carbamazepine [58], chloroquine [59], sulphadiazine [60], and chloramphenicol [61] has been reported to decrease significantly in children with PEM when compared with healthy normal children. The decreased oral absorption was attributed to the morphological changes in the jejunum.

Parasitic infestation is a common finding in PEM [56,62]. The influence of intestinal parasitosis on drug absorption in children with PEM has not been explored. Inspite of hepatomegaly seen in PEM, liver function tests were reported normal [63,64]. However, ultrastructure studies of liver biopsies have shown fatty changes, abnormal rough endoplasmic reticulum and mitochondria and decreased peroxisomes [64-66] which were completely reversible after nutritional rehabilitation. Biotransformation of drugs occur mostly in the liver via the microsomal enzyme pathways, therefore hepatic drug metabolism may be impaired in PEM. The activity of bilirubin-uridyldiphosphate (UDP) enzyme involved in the hepatic metabolism of chloramphenicol and some other drugs, as well the hepatic clearance of chloramphenicol, has been reported to decrease significantly in children with PEM [67]. Similarly, the plasma level of paraxanthine; a metabolite of caffeine has been used to indirectly measure CYP1A2 (an important hepatic metabolising enzyme) activity after caffeine administration [68]. The CYP1A2 activity, as well as the hepatic clearance of caffeine, was significantly decreased in children with kwashiorkor [68].

### Changes in renal function

Renal function is a very important determinant of the pharmacokinetic action of many drugs, but the effects of malnutrition on renal function have not been studied extensively. A study that utilised serial measurements of inulin clearance to measure the renal function of children with PEM had shown that the glomerular filtration rate (GFR) and renal blood flow are diminished, particularly in the presence of dehydration, but revert to normal after nutritional rehabilitation [69]. However, the amount of time required for the GFR to return to normal was not determined. Other studies have utilised less precise measurements of renal function, such as creatinine clearance or serum creatinine to study the effects of PEM on renal function. The result was however similar to the previous study that utilised inulin [70]. The urine of children with PEM is generally free of protein, glucose and formed elements such as casts, red blood cells and epithelial cells. Their blood urea nitrogen and creatinine are normal, indicating that children with PEM do not have an established renal failure [71].

Despite lack of evidence of established renal damage in children with PEM, the oedema observed in marasmic-kwashiorkor and kwashiorkor has been attributed to the inability of the kidneys to adequately excrete excess fluid and sodium [69], as well as to the presence of hypoproteinaemia and aflatoxins [8]. The impact of malnutrition on GFR is most relevant for children receiving drugs primarily excreted by the kidneys, such as the penicillin and aminoglycosides. While some studies have shown that the renal clearance of cefoxitin [36] and penicillin [32,33] were significantly decreased in children with PEM, the clearance of gentamicin [72,73], amikacin [74], ethambutol [75] and streptomycin [35] were also decreased but not significantly when compared with clearance in normal healthy children and after nutritional rehabilitation. These are drugs that are primarily excreted by the kidney and none of them showed evidence of toxic plasma concentrations, although multiple dose studies were not performed. Similarly, the renal excretion mechanisms of these drugs and their relationship to the renal function of children with PEM were not studied. It appears that the pathophysiological alterations in the renal function of children with PEM would only cause slight retention of renally excreted drugs but not up toxic levels that is usually associated with renal failure [37].

The pharmacokinetics of methotrexate had been studied in undernourished and well nourished adult patients with cancer. The drug is primarily excreted by the kidneys; its elimination half-life was more prolonged in the malnourished than well nourished patients [76]. The author therefore suggested that, in malnourished patients, relative weight; instead of body surface area or body weight, was a more appropriate parameter to determine drug dosage and should be used for drug dosing. This suggestion may indeed hold for dosing of drugs primarily excreted in the kidneys of children with PEM. Murry et al [77] had further suggested future studies to evaluate drug dosage adjustments in malnourished patients based on their specific features, such as GFR.

### Changes in cardiovascular system

Children with severe PEM have a smaller and thinner heart and a lower stroke volume [78]. The inability of the kidneys to adequately excrete excess fluid and sodium in marasmic-kwashiorkor and kwashiorkor also adversely affects the heart. Thus, the circulation is overloaded more easily than usual. The cell membranes of the heart become leaky because of oxidative damage [78]. The number of Na-K pumps in the cell membrane is reduced so as to conserve energy and the remaining pumps work more slowly. Thus intracellular sodium accumulation and potassium leakages occur, leading to electrolyte and fluid imbalance [78].

Treatment practices that involve inappropriate fluid therapy may contribute to cardiac failure in PEM [78,79]. Circulatory insufficiency is associated with a prolonged circulation time and inadequate absorption and distribution of drugs and nutrients. Oedematous PEM (marasmic-kwashiorkor and kwashiorkor) are characterised by salt and water retention [71]. This may contribute to the heart failure observed in severe PEM [79] and ultimately affect the effective transportation of drug in the circulatory system. Fluid retention would cause expansion of the extracellular fluid volume and may increase the volume of distribution of water soluble drugs.

### Changes in endocrine function

Studies of hormonal influences on drug metabolism, pharmacokinetics, or pharmacodynamics are still lacking in human, especially in children with PEM. However, endocrine system may probably play a major role in drug metabolism in children. This is because hormones mediate the dramatic physical changes of growth and development, and also serve to coordinate metabolic events in diverse tissues [80].

In the acute phase of PEM, serum insulin levels are depressed and growth hormone (GH) elevated among kwashiorkor children [81]. GH values are also elevated in the marasmic children but the levels were much lower than those for the kwashiorkor children. While the GH concentration fell steadily, the insulin rose especially among the kwashiorkor children after nutritional rehabilitation [81]. The adrenal glands of PEM children are atrophic at autopsy but plasma cortisol concentrations were elevated and response to corticotrophin challenge was unaffected [25]. Cortisol binds to serum proteins; therefore, hypoalbuminaemia would cause increase free plasma level of cortisol which may contribute to the abnormal glucose tolerance and oedema seen in kwashiorkor and marasmic-kwshiokor. In animal models, adrenalectomy and alloxan-induced diabetes abolished the daily variation in drug metabolism as well as producing alterations in the basal levels of hepatic microsomal enzyme activity [82]. Human growth hormone (hGH) replacement therapy in deficient children has been reported to substantially prolong the half-life of amobarbital [83]. With a substantial increase in plasma GH levels of PEM children, it would be necessary to study metabolism of amobarbital and other drugs with similar properties in children with PEM.

Thyroid hormones have been shown to substantially affect drug elimination in children [84,85]. A study has shown that the serum triiodothyronine (T3) was decreased in children with PEM in the face of normal thyroxine (T4) and free thyroxine index (FTI) with a corresponding decreased metabolism of antipyrine [86-88]. The low serum triiodothyronine (T3) was attributed to a reversible defect in extra-thyroidal conversion of T4 to T3 [89], similar to the impairment of biotransformation of labelled testosterone and oestradiol in patients with anorexia nervosa [90]. Drug metabolism was restored to normality in the anorexic patients after T3 administration and nutritional rehabilitation. Interaction between thyroid hormones and commonly prescribed drugs has been well documented [91], resulting in augmentation or attenuation of the action of either compound. Studies in animals and cell cultures have shown that thyroid hormones play an important role in the constitutive expression of the CYP450 enzymes [92], thus potentially altering the metabolism and effects of a variety of drugs. P-glycoprotein is expressed in the major organs associated with drug absorption, distribution and elimination from the body (e.g. intestine, kidney, liver, skin and the blood-brain barrier). Expression of intestinal P-glycoprotein in humans also appears to be influenced by thyroid hormones [93].

## Conclusion

Physiological changes in children with PEM are associated with abnormal disposition of drugs which may necessitate drug dosage modifications. However, the relevance of the abnormalities to the clinical management of children with PEM has not been demonstrated conclusively. Also, the available evidence did not provide any indication for drug dosage modification in PEM. Considering the high mortality rate associated with PEM, inappropriate therapy may be a cause but this has not been proven. Given that drug retention in PEM could result in adverse events which may be masked by a constellation of symptoms and signs of PEM, we therefore advocate more studies on disposition of drugs in PEM; especially those involving accurate determination of dosages of drugs frequently used for their treatment.

## Competing interests

The authors declare that they have no competing interests.

## Authors' contributions

KAO conceived the study, did the literature review and drafted the manuscript. IOS provided additional materials for the review, contributed to the discussion and foolproof the manuscript. Both authors read the final draft of the manuscript and agreed to the contents.
